# Quality of Life Assessment in Multiple Myeloma Patients Undergoing Dose-Reduced Tandem Autologous Stem Cell Transplantation

**DOI:** 10.4084/MJHID.2011.057

**Published:** 2011-11-28

**Authors:** A. Khalafallah, K. McDonnell, H.U. Dawar, I. Robertson, D. Woods

**Affiliations:** 1 Launceston General Hospital, Tasmania, Australia; 2 Launceston Clinical School, University of Tasmania, Australia; 3 School of Human Life Sciences, University of Tasmania, Australia; 4 Tasmanian Palliative Care Service, Australia

## Abstract

Few studies exist that consider health-related quality of life (HR-QoL) in patients with multiple myeloma (MM) undergoing tandem autologous stem cell transplantation (TASCT). Eighteen patients with advanced MM who underwent dose-modified TASCT were enrolled in this study between March 2006 and March 2008. Patients <60 year old (10) received conditioning with melphalan 140 mg/m^2^ and patients who were ≥60 years (8) received 100 mg/m^2^. The median age was 57.5 years (range 35–69). We conducted the European Organization of Research and Treatment of Cancer (EORTC) QLQ-C30 and the QLQ-MY24 questionnaires via interviews at presentation, after each ASCT and thereafter every 3 months for 24 months. Mean global health measure improved from 3.44 before transplant to 4.50 (1=very poor, 7=excellent) at the second and subsequent follow-up visits (P<0.001) and the mean global quality of life score improved from 3.61 to 4.71 (P<0.001). Pain symptoms were reduced (P=0.001), and physical functioning improved (P<0.001) throughout the period of post-transplant follow-up. Our study showed that dose-reduced TASCT is well tolerated with low toxicity albeit the transient reduction in QoL during both transplants. Post-transplant follow-up showed significant improvement in overall HR-QoL that reflects positively on the overall disease-outcome. Furthermore, a sole focus on patient-survival does not adequately provide indication regarding the tolerability and effectiveness of a proposed treatment on the patient’s perceived quality of life. As clinicians, our primary concern should be toward patient-welfare as well as survival. Therefore, we should employ the tools of QoL in conjunction with overall survival in order to deliver the best possible patient outcomes. The EORTC-QLQ-MY24 is a practical tool in measuring QoL in myeloma patients.

## Introduction

Multiple myeloma (MM) is a chronic incurable disease that is associated with reduced quality of life. This is in part due to chronic pain associated with osteolytic lesions, recurrent infections due to their immunocompromized status in addition to the side effects of different lines of treatment used to control this disease including autologous stem cell transplantation (ASCT).^1^–^4^

ASCT is a well recognized standard therapy for advanced myeloma disease with variable toxicities and considerable effects on quality of life.^5^–^8^ The use of melphalan high-dose therapy (200 mg/m^2^) presents significant side effects and subsequently a reduction of quality of life mainly due to infections, mucositis, increased use of blood products and prolonged stay in hospital.^7^–^8^

There are few studies that have analyzed health-related quality of life (HR-QoL) in conjunction with outcome and prognosis in patients with myeloma, who undergo ASCT.^9^–^11^ The European Organization for Research and Treatment of Cancer (EORTC) Quality of Life Group’s questionnaire for evaluating quality of life in cancer patients has been used in over 3000 international clinical trials. The latest (3^rd^) version, the EORTC QLQ-C30, was employed in this study, supplemented by the myeloma-specific module, the QLQ-MY24 after special permission from the European Organization of Research and Treatment of Cancer.^10^–^11^

We prospectively studied 18 Caucasian patients with multiple myeloma (MM) who were planned to receive dose-reduced melphalan followed by autologous tandem stem cell transplantation with three months between each transplant. The aim of the study is to assess HR-QoL in these patients in conjunction with the outcome of their disease as well as overall survival. We assessed the participants HR-QoL before the transplant to evaluate the impact of symptomatic myeloma on QoL, and subsequently, after each transplant periodically every 3 months for 24 months, to study the effect of dose-reduced ASCT and its prognostic significance on various dimensions of QoL.

## Patient and Methods

We studied 18 patients diagnosed with multiple myeloma (MM) according the World Health Organization (WHO) criteria, who underwent tandem ASCT during the period from March 2006 to March 2008.

The trial was approved by the Tasmanian Human Research Ethics Committee, Australia. An informed written consent was obtained from all participants according to code of Ethics. The study was registered in the Australia and New Zealand Clinical Trial Registry (ANZCTR) website http://www.ANZCTR.org.au/ACTRN12609000595213.aspx.

The tandem transplants were performed 3 months apart with dose-reduced melphalan, 100 mg/m^2^ for elderly patients ≥60 years and 140 mg/m^2^ for those <60 years of age followed by stem cell rescue in order to minimize the transplant associated toxicities. All patients presented during the study period with advanced MM according to the WHO criteria and undergoing tandem-ASCT were recruited in this prospective study. The male to female ratio was 16:2. The median age at first transplant was 57.5 years with an age range of (35–69). The patients were followed up for a median period of 35 months post transplant (range 29–49). A full patient profile was collected, including demographic and medical data and risk factors for multiple myeloma. Two patients succumbed at 22 and 26 months post transplant. The median time of follow up after diagnosis was 47.5 months (Range 35–81) (Table 1).

HR-QoL was measured as baseline at start of treatment before the transplant to evaluate the impact of symptomatic myeloma on QoL, and subsequently after each transplant. Thereafter HR-QoL was assessed quarterly for 24 months period to study the effect of disease and ASCT and its prognostic significance on various dimensions of QoL. We hypothesized that the interval between QoL measurements of three month should be adequate to record changes in QoL. This gives an adequate time for the patients to observe and record the changes of their QoL without a longer recall period that may substantiate unnecessary bias. Our study utilizes version 3.0 of the EORTC QLQ-C30 and the QLQ-MY24 (Multiple Myeloma Module) questionnaires with permission from the EORTC, presented longitudinally to a sample of Australian MM patients pre- and post-ASCT.^10^–^11^

### Methods

The EORTC quality of life questionnaire (QLQ) is an integrated system for assessing the HR-QoL of cancer patients participating in international clinical trials. The core questionnaire, the QLQ-C30, is the product of more than a decade of collaborative research. Following its general release in 1993, a large number of research groups have used the EORTC QLQ-C30 and the QLQ-MY24 in a wide range of oncology clinical trials; additionally, it has been used in various other non-trial studies. The EORTC QLQ-C30 is composed of functional and physical assessment as well as the global health measure. It covers general aspects of health-related quality of life and additional disease or treatment-specific questionnaire modules.^10^–^11^ Raw EORTC Quality of Life QLQ-C30 and the QLQ-MY24 questionnaires data were linearly converted to give standard scores into scales with ranges from 0 to 100 for each of the scales and single items. Accordingly, in the QoL scale; 0 represents the worst functioning quality of life and 100 the best quality of life. While in the symptoms scales; 0 represents absence of symptoms and 100 represents maximum presence of symptoms.

The myeloma module of EORTC QLQ-C30 (QLQ-MY24) that has been used here is designed for patients with MM to assess the symptoms and side effects of treatment and their impact on everyday life.^10^ The module comprises 24 questions addressing four domains of QoL important in myeloma: a pain scale, treatment side effects, social support and future perspective. The module was developed according to the guidelines and approved after formal review.^10^,^11^

The EORTC Quality of Life QLQ-C30 and the QLQ-MY24 questionnaire (myeloma specific module) was presented to all patients at interview before, during and after each transplant and regularly thereafter every 3 months in the first 24 months post transplant, by a research assistant who underwent special training with the specific QLQ-C30 and QLQ-MY24 modules.

### Statistical Analysis

EORTC Quality of Life QLQ-C30 and the QLQ-MY24 scales are rank-order data (ordered data but without fixed interval sizes across their range). Estimation of the changes of the data over time was performed using repeated-measures in ordinal logistic regression of those scale data over time: 1) each measurement point was compared to the initial measurement without assuming linearity of time; and also 2) the association of each measurement with time was assessed by non-linear regression using a 2^nd^-order polynomial model (time and time-squared in order to determine whether changes during treatments were reversed in the follow-up period). Results were expressed as: 1) odds ratios with 95% confidence intervals and p-values corrected for multiple comparisons by the Holm method; and 2) p-values for non-parametric trend for time (T) and time-squared (T^2^). Rate of mortality was estimated by the Mantel-Cox method (and time-to-event graphs for mortality were constructed) using death, loss to follow-up and completion of 48 months follow-up as censoring times. All analyses were performed using Stata/IC 10.1 for Windows (StataCorp LP, College Station, Tx, USA).

## Results

Physical, social, emotional, role and cognitive functions were analyzed and scored to four groups as those with severe, moderate, mild and no dysfunction. The gastrointestinal symptoms; loss of appetite, nausea and vomiting, constipation and diarrhea were similarly classified as were dyspnea, pain, fatigue and insomnia. The financial disability to the patient was analyzed as was the Global Health Measure (GHM) and the Global Quality of Life (GQL) Score.

### Progress of Symptoms, Functioning and Qol Scales Before, During and After ASCT

**Figure 1:**
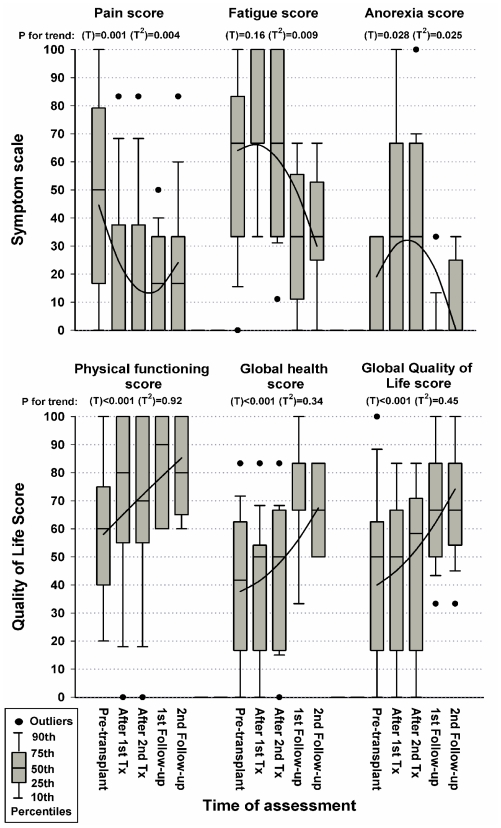
Percentile distribution of symptom scores (pain, fatigue and loss of appetite) and quality of life scores (physical functioning, global health and global Quality of Life) at different times of treatment with tandem autologous stem cell transplants in 18 patients treated for multiple myeloma. P-values for trend were estimated by repeated-measures, second-order polynomial {time (T) and time^2^ (T^2^)}, ordinal logistic regression. Trend lines were estimated by repeated-measures, second-order polynomial, general linear modeling for illustrative purposes only. In the QoL scale; 0 represents the worst functioning quality of life and 100 the best quality of life, while in the symptoms scales; 0 represents absence of symptoms and 100 represents maximum presence of symptoms.

Changes in symptom levels were summarized in Figure 1. The dominant symptoms present prior to the first ASCT were pain and fatigue. During the peri-treatment period there was a reduction in pain scores (OR 0.11; 95% CI 0.03 to 0.43; P for trend T=0.001 T^2^=0.004), but levels of fatigue increased (OR 2.97; 95% CI 1.05 to 8.38; T=0.16 T^2^=0.009). Levels of anorexia (OR 7.15; 95% CI 1.98 to 25.9; T=0.028 T^2^=0.025), nausea and vomiting (OR 3.12; 95% CI 0.84 to 11.5; T=0.08 T^2^=0.03), abdominal discomfort (OR 3.57; 95% CI 1.07 to 11.9; T=0.06 T^2^=0.045) as well as insomnia (OR 6.92; 95% CI 0.82 to 58.1; T=0.09 T^2^=0.08) increased during treatment. In addition, there was a trend to rise in dyspnea and diarrhea symptoms, but these did not reach statistical significance in the patients observed. All the worsening of symptoms that occurred during transplant were reversed at the first and second 3-monthly follow-up assessments and remained improved without change in subsequent follow-ups. The fatigue also improved compared to pre-treatment levels (OR 0.16; 95% CI 0.04 to 0.70; P=0.05), pain improved at substantially lower levels (OR 0.18; 95% CI 0.06 to 0.54; P=0.005), and other symptoms returned to their apparently low pre-transplant levels (Table 2).

Changes in levels of functioning and global health and quality of life measures were also described in Figure 1. Physical (OR 6.78; 95% CI 2.81 to 16.4; P for trend T<0.001) and social functioning (OR 3.16; 95% CI 1.42 to 7.02; T=0.021 T^2^=0.009) were moderately improved in the follow-up period, as well as the patient’s assessment of their global health status (OR 7.99; 95% CI 2.67 to 23.9; P for trend T<0.001) and quality of life (OR 7.06; 95% CI 2.74 to 18.2; P for trend T<0.001) and remained the same in the 3 monthly follow up. Of interest, patients reported increased financial difficulties during transplant, and also somewhat reduced social functioning, which recovered during follow-up assessments. Role, cognitive and emotional functioning appeared to be unaltered throughout the assessment periods. There was a steady rise in physical functioning, mirrored by a steady rise in global health status and quality of life. Furthermore, it seems that the psychosocial dimensions of QoL did not change with the alterations of disease-status during the period of follow up; however, physical QoL and global QoL were closely linked and affected by disease-status (Table 2).

### Patient Survival Following Diagnosis of Multiple Myeloma

**Figure 2:**
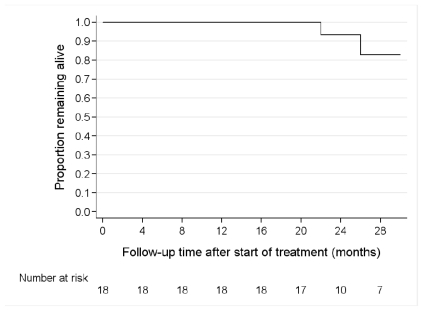
Overall survival in the months following treatment with dose-reduced tandem ASCT in 18 patients treated for multiple myeloma

Patients were followed-up for a median period of 35 months (median; range 29 to 49 months). Two patients died at 22 and 26 months, with an estimated mortality rate of 5.3% per year (95% confidence intervals 1.3 to 21.1 % per year). After a median follow up period of 35 months, 16 patients were alive and 16 remained in remission; 4 in complete remission (CR), 12 in partial remission (PR), while 2 patients experienced further disease progression and succumbed at 22 and 26 months of follow up post-transplant (Figure 2).

## Discussion

Our study provided further evidence of the significant impairment of HR-QoL in patients with multiple myeloma at onset of therapy.^12^–^14^

In our series, the main symptoms prior to ASCT that myeloma patients presented with were pain and fatigue, while during the peri-treatment period there was a reduction in pain scores with increased fatigue. This is largely due to the direct effect of the intermediate to high-dose chemotherapy on the hemoglobin levels causing a transient anemia. In contrast, the pain scores improved due to several factors including effective control of the disease in addition to employing pain medications during the active treatment period (p=0.004). Furthermore, levels of nausea, vomiting, abdominal discomfort and insomnia increased temporarily during ASCT, mostly due to the adverse effect of intermediate to high dose melphalan. All the worsening of symptoms that occurred during the transplant were reversed at the first or second 3-monthly follow-up assessment and remained stable in subsequent follow-ups. Moreover, the level of fatigue improved compared to pre-transplant levels (p=0.05) with the pain remaining at substantially significant lower levels (p=0.006) compared to the pre-transplant period. Other symptoms returned to their pre-transplant levels.

Physical and social functioning was moderately improved in the 3 monthly follow-up period, as was the patient’s assessment of their global health status and quality of life. Interestingly, MM patients reported increased financial difficulties during the ASCT period (p=0.07) as well as reduced social functioning (p=0.05). However, this improved during follow-up assessments. There was a steady rise in physical functioning, reflected by a steady rise in global health status and quality of life (p=0.001). Role, cognitive and emotional functioning appeared to be unaltered throughout the assessment periods. We observed that the psychosocial dimensions of QoL were found to be independent factors, while physical QoL and global QoL were affected by disease progression. Furthermore, our data showed a good correlation between physical QoL scales such as pain, fatigue, physical functioning and global QoL than between psychosocial dimensions such as role, emotional, social, and cognitive functioning in accordance with other studies with improved QoL measures in our series compared to the standard high-dose ASCT.^15^–^17^ A multivariate analyses by Prieto and colleagues revealed that higher systemic symptomatology scores were significantly associated with impaired overall quality of life with a higher psychosocial distress in patients undergoing stem cell transplantation.^20^ However, in the new era of novel agents for treatment of MM such as proteasome inhibitor; bortezomib and the immunomodulatory agents; thalidomide and lenalidomide, there is a significant improvement of quality of life in the management of older patients and/or those not eligible for transplantation.^21^

Our study has a few shortcomings. First, it has a relatively small number of patients; therefore it is difficult to draw a concrete conclusion, however, due to lack of data in this regard, it is worthwhile documenting such results. Second, owing to the inherent difficulties in measuring quality of life, there is no dedicated control group for patients who have received a single transplant with high dose melphalan, so that the very few published data from other cohorts are being used as the point of reference.^20^

## Conclusions

Our analysis shows that dose-modified tandem ASCT is well tolerated with acceptable toxicity and side effects albeit the transient impairment in QoL during both transplants. Nevertheless, the post transplant follow up showed significant improvement in the quality of life that certainly reflects positively in the overall disease outcome. Furthermore, the present study confirmed the EORTC QLQ-C30 with the QLQ-MY24 questionnaires to be a practical instrument in measuring the QoL in patients with advanced myeloma disease both before and after treatment. After a median follow up of 35 months, 16 patients were alive and remained in remission (4 CR, 12 PR).

Clearly, a sole focus on patient’s survival does not necessarily reflect the tolerability and effectiveness of a proposed treatment on the patient’s perceived quality of life.^19^,^20^ Therefore, measures of overall survival should be employed in conjunction with the impact on health-related quality of life to achieve the best possible outcomes.

## Figures and Tables

**Table 1:** Patient characteristics.

No. of patients	18

Median age (range)	57.5 (35–69)
>60	8
<60	10

Sex (M : F)	16 : 2

Stage of myeloma disease according Durie & Salmon	
IIIA	17
IIIB	1

Type of myeloma:	
IgG	12
IgA	2
Light Chain	4

Transplant	
Upfront therapy	16
After achieving remission in a relapsed disease	2

Chemotherapy regimen prior Tx	
VAD	3
Dexamethasone and Thalidomide	9
Velcade	4
Lenalidomide	2

Time followed up since diagnosis (Months)	47.5 (35–81)

Time followed up since First transplant (Months)	35 (29–49)

High risk disease; stage III with hypercalcaemia, renal failure, high B_2_ Microglobulin	8
High cytogenetic risk (e.g. 13q-, 17p-, t(4,14))	6
Patients with stage III disease with standard risk factors	4

Outcome of the disease	
Alive	16
Complete remission	4
Partial remission	12
Deceased due to disease progression	2

**Table 2:** Progress of functioning and quality of life in patients receiving tandem autologous stem cell transplantation for multiple myeloma

EORTC QLQ-C30 Scale^1^	Mean (SD)	Mean Difference^2^	OR^3^	95% CI	P-value^4^
**Physical functioning**					
Pre-transplant	57.5 (25.2)		1.00		(T:<0.001)
After 1^st^ Tx	67.8 (30.8)	10.3	2.48	(0.78 to 7.89)	0.25
After 2^nd^ Tx	66.7 (30.7)	9.2	2.22	(0.77 to 6.36)	0.14
1^st^ Follow-up	83.8 (18.2)	26.3	6.78	(2.81 to 16.4)	<0.001
2^nd^ and subsequent Follow-up	83.8 (16.7)	26.3	6.40	(2.00 to 20.5)	0.005

**Role functioning**					
Pre-transplant	56.3 (25.0)		1.00		(T:0.38 & T^2^:0.68)
After 1^st^ Tx	52.8 (36.3)	−3.5	0.84	(0.26 to 2.77)	>0.9
After 2^nd^ Tx	52.8 (40.1)	−3.5	0.86	(0.24 to 3.05)	0.81
1^st^ Follow-up	90.6 (20.2)	34.4	12.43	(3.45 to 44.8)	0.001
2^nd^ and subsequent Follow-up	65.6 (23.9)	9.4	1.70	(0.74 to 3.90)	0.63

**Cognitive functioning**					
Pre-transplant	72.9 (28.5)		1.00		(T:0.43 & T^2^:0.47)
After 1^st^ Tx	66.7 (23.6)	−6.3	0.52	(0.19 to 1.46)	0.86
After 2^nd^ Tx	67.6 (21.0)	−5.3	0.56	(0.19 to 1.62)	0.86
1^st^ Follow-up	72.2 (20.6)	−0.7	0.81	(0.39 to 1.70)	0.58
2^nd^ and subsequent Follow-up	67.7 (32.5)	−5.2	0.71	(0.31 to 1.64)	0.84

**Emotional functioning**					
Pre-transplant	81.3 (27.1)		1.00		(T:0.91 & T^2^:0.96)
After 1^st^ Tx	83.8 (19.7)	2.5	0.88	(0.24 to 3.23)	>0.9
After 2^nd^ Tx	83.8 (19.9)	2.5	0.95	(0.25 to 3.58)	0.94
1^st^ Follow-up	92.2 (13.5)	11.0	2.13	(0.79 to 5.76)	0.55
2^nd^ and subsequent Follow-up	83.9 (26.4)	2.6	1.27	(0.52 to 3.11)	>0.9

**Social functioning**					
Pre-transplant	62.5 (15.5)		1.00		(T:0.021& T^2^:0.009)
After 1^st^ Tx	51.9 (27.3)	−10.6	0.42	(0.17 to 1.03)	0.18
After 2^nd^ Tx	52.8 (26.4)	−9.7	0.45	(0.19 to 1.07)	0.14
1^st^ Follow-up	65.6 (25.6)	3.1	1.25	(0.35 to 4.44)	0.73
2^nd^ and subsequent Follow-up	76.0 (18.2)	13.5	3.16	(1.42 to 7.02)	0.019

**Financial difficulties**					
Pre-transplant	29.2 (29.5)		1.00		(T:0.008 & T^2^:0.003)
After 1^st^ Tx	48.1 (38.3)	19.0	2.55	(0.93 to 6.96)	0.21
After 2^nd^ Tx	48.1 (38.3)	19.0	2.55	(0.93 to 6.96)	0.21
1^st^ Follow-up	26.7 (31.4)	−2.5	0.84	(0.30 to 2.38)	0.74
2^nd^ and subsequent Follow-up	12.5 (24.0)	−16.7	0.28	(0.10 to 0.81)	0.077

**Global health status**					
Pre-transplant	38.5 (25.6)		1.00		(T:<0.001)
After 1^st^ Tx	40.7 (23.7)	2.2	1.17	(0.40 to 3.40)	0.78
After 2^nd^ Tx	45.4 (24.8)	6.8	1.81	(0.61 to 5.33)	0.57
1^st^ Follow-up	61.1 (23.3)	22.6	5.51	(1.30 to 23.3)	0.061
2^nd^ and subsequent Follow-up	65.6 (15.5)	27.1	7.99	(2.67 to 23.9)	0.001

**Global QoL**					
Pre-transplant	41.7 (29.2)		1.00		(T:<0.001)
After 1^st^ Tx	43.5 (27.5)	1.9	1.22	(0.47 to 3.20)	0.68
After 2^nd^ Tx	48.1 (30.7)	6.5	1.77	(0.66 to 4.74)	0.51
1^st^ Follow-up	71.1 (20.4)	29.4	7.12	(2.23 to 22.7)	0.003
2^nd^ and subsequent Follow-up	70.8 (18.8)	29.2	7.06	(2.74 to 18.2)	<0.001

EORTC Quality of Life Functioning Scales: each have a range of 0–100 with 0 being worst functioning and 100 best functioning. Mean difference QoL scale scores at specified times compared to initial pre-transplant assessment, estimated by general linear modelling (GLM) corrected for repeated measures (this treats the scale scores as continuous interval data, which is not appropriate, but shown for illustrative purposes only). Comparison of QoL scale scores at specified times compared to initial pre-transplant assessment, estimated by ordinal logistic regression (OLR) corrected for repeated measures and expressed as odds ratio with 95% confidence intervals and P-values (OLR is a non-parametric version of GLM that treats the scale scores as rank order data). P-values shown are: 1) in the Pre-transplant row, the P-value for 2^nd^-order polynomial trend (T=time, and T^2^=time-squared) demonstrating (or not) the reversal of the effects of the receiving dual autologous stem cell transplant; and 2) in subsequent rows, the comparisons of the other time periods to the pre-transplant assessment corrected for multiple comparisons by the Holm method (against the OR 1.00, all estimated by OLR which does not assume linearity of time)
